# Updating Nutritional Data and Evaluation of Technological Parameters of Italian Milk

**DOI:** 10.3390/foods2020254

**Published:** 2013-06-20

**Authors:** Pamela Manzi, Maria Gabriella Di Costanzo, Maria Mattera

**Affiliations:** Agricultural Research Council-Research Centre for Food and Nutrition (C.R.A.-NUT), Via Ardeatina 546, Roma 00178, Italy; E-Mails: dicostanzo@inran.it (M.G.D.C.); mattera@inran.it (M.M.)

**Keywords:** commercial milk, technological treatment, minerals, choline, fat-soluble vitamins, cholesterol, color measurement

## Abstract

Different technologically treated Italian milks (whole and semi-skimmed ultra-high temperature (UHT), pasteurized and microfiltered milk), collected from 2009 to 2012, were evaluated for nutritional and technological properties. No significant differences in calcium and sodium were detected (*p* > 0.05), while significant differences were observed concerning phosphorus content, between whole and semi-skimmed milk, and lactose content, between pasteurized and UHT milk (*p* < 0.05). In UHT milk, lactose isomerization occurred, and lactulose (from 8.6 to 104.0 mg/100 g) was detected. No significant differences (*p* > 0.05) were detected for choline, a functional molecule, between whole (11.3–14.6 mg/100 g) and semi-skimmed milk (11.1–14.7 mg/100 g), but there were significant differences (*p* < 0.05) in processing milk (UHT *vs.* pasteurized milk and UHT *vs.* microfiltered milk). Among the unsaponifiable compounds, only 13 *cis* retinol and *trans* retinol showed differences in technologically treated milk (pasteurized *vs.* UHT milk and microfiltered *vs.* UHT milk; *p* < 0.05). In this research, the greater was the “severity” of milk treatment, the higher was the percent ratio 13 *cis*/*trans* retinol (DRI, degree of retinol isomerization). The degree of antioxidant protection parameter (DAP), useful to estimate the potential oxidative stability of fat in foods, was significantly different between whole and semi-skimmed milk (*p* < 0.05). Finally, the evaluation of color measurement of whole milk showed a good correlation between beta carotene and b* (*r* = 0.854) and between lactulose and a* (*r* = 0.862).

## 1. Introduction

Milk contains the main nutrients, such as fats, proteins, carbohydrates, minerals and vitamins, necessary to the early life stages: the high nutritional quality of milk facilitates to achievement of individuals’ nutritional daily requirements.

In 2011, the world cow milk production was nearly 606 million tons, and cow milk dominated the global milk production (84%). Consumption of milk and dairy products varies from country to country: in 2012, the per capita consumption of milk slowly increased in the world: particularly, it increased in South America and Asia and decreased in Europe and Oceania [1,2,3]. In Italy, in detail, the per capita consumption of milk in 2011 was 53 kg, and in 2012, the production of milk achieved 2,344,330 tons (3.8% less than 2011). 

In Italy, since 2007, raw milk can be formally commercialized by self-service automatic vending machines [4], and an increasing number of people consume raw milk, probably due to the low cost of this item. Nevertheless, numerous epidemiological studies showed that raw milk can be contaminated by a variety of pathogens, some of which are associated with human disease and pathogenic microorganisms, such as *Listeria*, *Campylobacter*, *Salmonella* spp. and *Escherichia coli*, which were isolated in raw milk [5,6,7,8]. In Italy, raw milk can be sold only at the farms, but the expiration date must not be more than three days from the production. Moreover, a warning label “raw milk must be boiled before consumption” has to be displayed on the automatic vending machines [4]. Several technological treatments are commonly used in order to eliminate the pathogens or to slow their growth and to preserve milk for longer periods of time. Among processed milks, UHT (ultra-high temperature), pasteurized and microfiltered milk, are widely consumed in Italy. 

Among heat treatments, pasteurization, a relatively mild heat treatment (at least 71.7 °C for 15 s), is sufficient to destroy disease-causing microorganisms: pasteurized milk shows a negative reaction to the phosphatase test and a positive reaction to the peroxidase test, and it must be preserved at ≤6 °C. Pasteurization is used to extend the shelf-life of milk for six days, while UHT treatment, obtained by applying heat at high temperature (>135 °C) for a short time (at least 1 s), permits milk to be held for a long period (90 days) at room temperature before being used [9]. This “severe” treatment destroys all residual spoilage microorganisms and their spores, in order to prolong milk shelf life considerably. 

Furthermore, microfiltration is a technique applied to milk for bacteria removal: this is a membrane separation processes useful for extending the shelf life of milk, maintaining its organoleptic and nutritional properties unchanged as much as possible. In the microfiltration process, milk cream is centrifugally removed from whole milk, skim milk is microfiltered through a membrane with pore sizes ranging from 1.4 to 2 μm in order to remove bacteria from the fluid permeate. After, this treatment permeate and cream are pooled, and lastly, the milk is pasteurized [10]. The use of microfiltration in the dairy industry is a powerful tool for the improvement of hygienic properties with a mild heat treatment of milk [11,12,13]. Milk composition has a dynamic nature, and it is influenced by many features: its composition differs from season, climate, feeding management, stage of lactation, energy balance and the health status of cows [14]. Moreover, it is well known that modifications could be also related to technological treatments: during heat treatments, numerous reversible and irreversible changes in chemical composition and in physical and organoleptic characteristics of milk can occur, according to the applied temperature [15,16].

From a nutritional point of view, it is important to take into account the effect of technological treatment on milk: for instance, cow’s milk and other dairy foods are the major source of calcium, but a negative effect on calcium solubility was observed after *in vitro* digestion of overheated milk compared with UHT milk [17]. Moreover, protein denaturation, with particular regard to whey protein solubility, Maillard’s reaction and isomerization of lactose lead to a decrease in the nutritional value of milk with modification of the organoleptic properties (cooked taste and browning).

Therefore, the aim of this study was to update and extend information on the nutrient content of retailed milk in Italy; this survey was performed from 2009 until 2012.

In particular, the chemical composition (minerals, lactose, fat soluble vitamins, cholesterol), the nutritional properties (choline, lactulose), color and quality parameters of commercial Italian cow milk were assessed to evaluate and compare different technologically (UHT, pasteurized and microfiltered milk) treated milk, either whole or semi-skimmed. 

## 2. Experimental Section

### 2.1. Samples

Commercially available, different brands of cow’s milk were collected from Italian grocery stores and supermarkets. All the samples were stored as indicated on the label prior to testing (5 °C for pasteurized and microfiltration milk; room temperature storage for UHT milk). All the details of the analyzed samples are reported as follows:

- 18 samples of whole milk: 3 pasteurized (PW), 3 microfiltered (MW) and 12 UHT (UHTW) milks;
- 20 samples of semi-skimmed milk: 2 pasteurized (PS), 4 microfiltered (MS) and 14 UHT (UHTS) milks.

### 2.2. Chemicals

All reagents used were of HPLC grade or at least of the highest purity available. Standards were obtained from Sigma Aldrich (St Louis, MO, USA) and Merck KGaA (Darmstadt, Germany). Ultrapure water, of the grade required for critical laboratory applications, such as HPLC mobile phase preparation, was prepared by an ion exchange system to >18 mΩ resistivity (Millipore, MA, USA).

### 2.3. Equipments

The following was used: a spectrophotometer (Shimadzu, Model: 1800, Tokyo, Japan); a handheld tristimulus colorimeter (Konica Minolta CR-400, Minolta Limited, Milton Keynes, UK); an atomic absorption spectrometer (Perkin Elmer Model: A.Analyst 300, Norwalk, CT, USA) and an HPLC analytical system, Alliance (Waters model: 2695, Milford, MA, USA), with a fluorescence detector (Waters model: 2475, Milford, MA, USA), a UV-vis detector (Waters model: 2487, Milford, MA, USA) and a detector refractive index (Waters model: 2414, Milford, MA, USA).

### 2.4. Minerals Determination

The samples were analyzed after ashing: briefly, 2 g of milk were weighed into platinum crucibles and ashed in the furnace at 525 °C for 16 h. Calcium and sodium were determined using an atomic absorption spectrometer, and phosphorus was measured at 400 nm by spectrophotometer [18].

### 2.5. Lactose and Lactulose

Two-point-five grams of milk samples were dissolved in warm water (15 mL; about 40 °C) and, in order to obtain the precipitation of fat and protein, 0.25 mL of Carrez I (potassium hexacyanoferrate II: 3.6 g/100 mL water) and Carrez II reagents (zinc acetate dihydrate: 7.2 g/100 mL water) were added sequentially by mixing, according to the procedure of Indyk *et al*. [19]. The extract was made up to volume (25 mL), filtered through filter paper (Whatman no. 541, Maidstone, England) and further passed through a 0.45 μm membrane. The clear filtrate was injected into the HPLC system: in the chromatographic procedure [20], two Pinnacle II Amino 3 μm 150 × 4.6 mm (i.d.) columns were connected in series, with a Pinnacle II Amino Guard Cartridge precolumn (Restek, Bellefonte, PA, USA) and maintained at 35 °C. The mobile phase was CH_3_CN:H_2_O 75:25 (v/v) with a flow rate 1.0 mL/min, and the refractive index detector was maintained at 35 °C.

### 2.6. Choline

The enzymatic-spectrophotometric method was performed according to the Association of Official Analytical Chemists (AOAC) method [21]. To 20 mL of milk, 3 M HCl were added to perform a traditional hydrolysis for 3 h at 70 °C in order to release the majority of the predominantly bound choline. After the hydrolysis steps, the samples were cooled, and then, the pH value was adjusted to 3.5–4.0 with a 10 M NaOH solution. Residual choline phospholipids were cleaved with phospholipase D and free choline incubated with choline oxidase, with liberation hydrogen peroxide. In the presence of peroxidase, a quinoneimine chromophore was formed with 4-aminoantipyrine. Absorbance was measured at 505 nm, as choline hydroxide.

### 2.7. Unsaponifiable Components and Tracing Parameters

To determine α-tocopherol, β-carotene, 13 *cis* retinol, *trans* retinol and cholesterol, milk samples were subjected to alkaline digestion (70 °C for 30 min) with 2 mL KOH, 2 mL ethanol, 1 mL NaCl (1%) and 5 mL ethanolic pyrogallol (60%) and extracted according to the method of Panfili *et al*. [22]. The suspension was extracted twice with 15 mL hexane: ethyl acetate (9:1, v/v) and the organic phase collected and evaporated to dryness. The residue was dissolved in 2 mL mobile phase (2-propanol 1% in *n*-hexane), injected and analyzed by normal phase HPLC [22]. In the chromatographic procedure, a Phenomenex, Kromasil, 5 µm Si 250 × 4.6 mm and a fluorescence detector and UV-vis detector connected in series were utilized to determine β-carotene (450 nm), cholesterol (208 nm), α-tocopherol (excitation 280 nm, emission 325 nm); *13 cis* retinol and *trans* retinol (excitation 325 nm, emission 475 nm).

DRI parameter (degree of retinol isomerization): calculated as the percent ratio of *13 cis* retinol/*trans* retinol [23].

DAP parameter (degree of antioxidant protection): the molar ratio of antioxidant compounds to a selected oxidation target [24]. In milk and dairy products, the antioxidant compounds considered were α-tocopherol and β-carotene, and the oxidation target molecule was cholesterol.

### 2.8. Color Measurements

Color measurements of the whole milk samples were determined by a handheld tristimulus colorimeter. Color measurements were performed using a CIE (Commission Internationale de L’Eclairage) standard D65 illuminate, an angle of observation of 0° and an 8 mm diameter field of view. The colorimeter was calibrated for light source with a white calibration plate before color measurements were taken. In the L*a*b* color space (CIELAB space), the L*, a*, b* coordinates were measured: L*, indicating lightness, and a* and b*, the chromaticity coordinates.

### 2.9. Statistical Analysis

Measurements were made in triplicate, and the statistical treatment of data (ANOVA coupled with the Tukey’s *post hoc* test and Student’s *t*-test) was performed using the KaleidaGraph 3.6 software (Synergy Software, Reading, PA, USA). *p*-Values <0.05 were considered significant.

## 3. Results and Discussion

### 3.1. Mineral Content

Milk is considered as a good source for protein, fat and major minerals: all these nutrients are important for growth and health. The mineral fraction of milk, in particular, comprises cations and anions, and some of them are considered relatively constant, even if slight variations can be observed [25] in calcium (104.3–128.3 mg/100 g), phosphorus (93.0–99.2 mg/100 g), sodium (39.1–64.4 mg/100 g) and potassium (121.2–168.1 mg/100 g).

In Table 1, the contents of calcium, phosphorus and sodium in all the studied samples were shown: calcium ranged from 101.5 to 123.4 mg/100 g, phosphorus from 86.6 to 102.6 mg/100 g, while sodium showed the wider interval (from 36.4 to 51.9 mg/100 g).

**Table 1:** Calcium, phosphorus and sodium content (mg/100 g) and molar ratio of Ca/P in whole and semi-skimmed commercial Italian milk. Data are shown as the mean with standard deviation (SD) and minimum and maximum values.

Milk		Calcium	Phosphorus	Sodium	Ca/P
*Whole—18 samples*					
	*Mean ± SD*	*112.7 ± 5.3*	*94.2 a ± 3.2*	*43.8 ± 3.6*	*0.9*
	*Min–Max*	*101.5–121.7*	*86.6–98.1*	*36.4–51.9*	*0.9–1.0*
	PW—3 samples											
	Mean ± SD	111.1 ± 1.8	93.3 ± 0.6	41.5 ± 5.0	0.9
	Min–Max	109.0–112.2	92.9–94.0	36.3–46.3	0.9–0.9
	MW—3 samples											
	Mean ± SD	110.9 ± 5.1	92.0 ± 4.6	44.5 ± 7.1	0.9
	Min–Max	105.1–114.2	86.6–95.1	37.8–51.9	0.9–0.9
	UHTW—12 samples											
	Mean ± SD	113.5 ± 6.0	95.1 ± 3.1	44.1 ± 2.2	0.9
	Min–Max	101.5–121.7	88.1–98.1	40.5–47.8	0.9–1.0
*Semi-Skimmed—20 samples*					
	*Mean ± SD*	*115.5 ± 4.9*	*96.5 b ± 3.4*	*43.3 ± 3.3*	*0.9*
	*Min–Max*	*105.1–123.3*	*88.8–102.6*	*38.7–52.0*	*0.9–1.0*
	PS—2 samples											
	Mean ± SD	114.6 ± 1.1	95.4 ± 0.1	45.9 ± 2.8	0.9
	Min–Max	113.8–115.3	95.4–95.5	43.9–47.8	0.9–0.9
	MS—4 samples											
	Mean ± SD	113.7 ± 4.5	95.1 ± 4.5	44.4 ± 5.4	0.9
	Min–Max	107.0–116.6	88.8–99.1	39.0–52.0	0.9–0.9
	UHTS—14 samples											
	Mean ± SD	116.2 ± 5.4	97.1 ± 3.3	42.6 ± 2.6	0.9
	Min–Max	105.1–123.4	91.3–102.6	38.7–47.0	0.9–1.0

PW = pasteurized whole milk; MW = microfiltered whole milk; UHTW = UHT whole milk; PS = pasteurized semi-skimmed milk; MS = microfiltered semi-skimmed milk; UHTS = UHT semi-skimmed milk; Values in the same column with different lowercase letters are significantly different (*p* < 0.05).

According to the data obtained, only phosphorus showed a difference (*p* < 0.05) between whole (94.2 ± 3.2 mg/100 g) and semi-skimmed milk (96.5 ± 3.4 mg/100 g). Phosphorus is linked to caseins and to phospholipids, mostly located in the globule membrane, but it is also in soluble and insoluble forms. Probably several factors, such as stage of lactation, nutritional status of animals or genetic factors [26], could affect the mineral content more. According to Hidiroglou and Proulx [27], in dairy cattle, animals exhibited distinct individual differences in their milk phosphorus content. Therefore, it is difficult to explain these differences between whole and semi-skimmed milk, overall, in commercially samples, because of phosphorous distribution in milk. 

Moreover, in the same Table 1, the molar ratio of calcium/phosphorus was shown. Dairy foods have long been accepted as an excellent source of calcium. Adequate intake of calcium and other nutrients from dairy product can help to lower the risk of high blood pressure, body weight/fat gain and colon cancer [28,29,30]. In childhood, overall, an adequate ratio of Ca/P (from 0.9 to 1.7) [31] is necessary. This ratio in the studied milk samples ranged from 0.9 to 1.0, and any differences were detected between whole and semi-skimmed milk or among different milk treatments (UHT *vs.* pasteurized *vs.* microfiltered milk).

### 3.2. Lactose and Lactulose Content

In Table 2, the amount of lactose in the studied commercial milks were shown. Lactose (*O*-β-d-galactopyranosyl-(1-4)-β-d-glucose) is the best known sugar, because of its abundance in milk; its biosynthesis takes place in the mammary gland. Lactose is a disaccharide, hydrolysed by lactase in the intestinal lumen to glucose and galactose: these two sugars are then transported across the brush border membrane of epithelial cells into the cytosol and distributed to the different tissues. Generally, mammals have high lactase activity in the early stages of their lives and low lactase activity when they reach adulthood, so lactose intolerance is a common problem in adults. The symptoms of lactose intolerance are abdominal pain, nausea and diarrhea. This is due to the intestine lacking the capability of absorbing disaccharides; therefore, undigested lactose serves as a fermentable substrate for the bacterial microflora [32,33]. Many microorganisms are able to use the lactose as their main carbon source and for growth, such as lactic acid bacteria, and dairy products, like yogurt or kefir, are the most common products obtained from milk through fermentation [33]. In human, the severity of symptoms of lactose intolerance varies, depending on the amount of lactose that everyone can tolerate: for all these reasons, in this work, the amount of lactose in commercial milk was evaluated.

**Table 2:** Lactose (g/100 g) and lactulose (mg/100 g) in whole and semi-skimmed commercial Italian milk. Data are shown as the mean with standard deviation (SD) and minimum and maximum values.

Milk		Lactose	Lactulose
*Whole—18 samples*			
	*Mean ± SD*	*4.8 ±* *0.2*	
	*Min–Max*	*4.3–5.3*	
	PW—3 samples		
	Mean ± SD	4.5 a ± 0.2	n.d.
	Min–Max	4.3–4.6	
	MW—3 samples		
	Mean ± SD	4.7 ± 0.1	n.d.
	Min–Max	4.6–4.8	
	UHTW—12 samples		
	Mean ± SD	4.9 b ± 0.2	43.3 ± 28.8
	Min–Max	4.6–5.3	10.2–90.6
*Semi-skimmed—20 samples*			
	*Mean ± SD*	*4.9* * ±* * 0.2*	
	*Min–Max*	*4.3–5.4*	
	PS—2 samples		
	Mean ± SD	4.9 ± 0.1	n.d.
	Min–Max	4.8–4.9	
	MS—4 samples		
	Mean ± SD	4.8 ± 0.1	n.d.
	Min–Max	4.6–4.9	
	UHTS—14 samples		
	Mean ± SD	4.9 ± 0.3	44.2 ± 35.4
	Min–Max	4.3–5.4	8.6–104.0

n.d. = not detectable (<LOD is 0.013 mg/mL); PW = pasteurized whole milk; MW = microfiltered whole milk; UHTW = UHT whole milk; PS = pasteurized semi-skimmed milk; MS = microfiltered semi-skimmed milk; UHTS = UHT semi-skimmed milk; Values in the same column with different lowercase letters are significantly different (*p* < 0.05).

The data (Table 2) showed no significant differences in lactose content between whole and semi-skimmed milk samples: lactose in whole milk ranges from 4.3 to 5.3 g/100 g and in semi-skimmed milk from 4.3 to 5.4 g/100 g. 

Significant differences in lactose content were observed in different technologically treated milk and, in particular, between pasteurized (4.5 ± 0.2 g/100 g) and UHT (4.9 ± 0.2 g/100 g) milk (*p* < 0.05). No definite conclusion can be drawn concerning these differences, because all the samples were collected from grocery stores and supermarkets: the variation in lactose content may be attributed to the quality of milk. According to Siddique *et al*. [34], UHT treatment exhibited a pronounced effect on lactose degradation, and commercial milk poor in quality showed lower lactose. According to some authors [35], actually, lactose was one of the most useful markers for monitoring serious diseases in dairy animals, such as mastitis. Moreover, in UHT milk, where milk is subject to severe heat processes, lactulose was detected (the lactulose content in UHT whole and semi-skimmed milk). In the condition of temperature of the UHT process, lactose isomerizes to lactulose, a meaningful chemical indicator for UHT or sterilized milk, but not effective for pasteurized milk [36]. According to several authors [37,38], directly heated UHT milk contained less lactulose than indirectly heated UHT milk, and sterilized milk contained more lactulose than both types of UHT milk. Many authors [37,38,39] showed the presence of lactulose in UHT and in-bottle sterilized milk, milk powder and spray-dried milk in an amount directly proportional to the process severity (industrial condition of time and heating). The obtained data showed no significant differences (*p* > 0.05) in whole and semi-skimmed UHT milk, and the contents varied considerably from 8.6 to 104.0 mg/100 g. It was very difficult to explain this huge range in lactulose content, because all the samples were collected from grocery stores and supermarkets, and it was not possible to make a distinction between directly heated UHT milks and indirectly heated UHT milks.

### 3.3. Choline Content

Among functional molecules, natural components with effects on well-being and health [40,41], the choline content was detected.

Choline, β-hydroxyethyltrimethyl-ammonium hydroxide, is an essential nutrient for many mammalian species. It has numerous functions: it is a phospholipid and a neurotransmitter acetylcholine precursor, it is effective in improving neurological disorders and memory strength and it is an important source of methyl groups [42,43,44,45,46].

The contents of choline (referred to as choline hydroxide) in the studied milk samples are shown in Table 3. The content of this compound ranged from 11.3 to 14.6 mg/100 g in whole milk and from 11.1 to 14.7 mg/100 g in semi-skimmed milk: according to these results, no significant differences (*p* > 0.05) were detected between whole and semi-skimmed milk.

**Table 3:** Choline hydroxide (mg/100 g) in whole and semi-skimmed commercial Italian milk. Data are shown as the mean with standard deviation (SD) and minimum and maximum values.

Milk	Choline hydroxide (mg/100 g)	
*Whole—18 samples*		
	*Mean ± SD*	*13.0 ± 1.0*
	*Min–Max*	*11.3–14.6*
	PW—3 samples	
	Mean ± SD	12.2 a ± 0.3
	Min–Max	11.8–12.4
	MW—3 samples	
	Mean ± SD	11.8 a ± 0.6
	Min–Max	11.5–12.5
	UHTW—12 samples	
	Mean ± SD	13.4 b ± 0.8
	Min–Max	11.3–14.6
*Semi-skimmed milk—20 samples*		
	*Mean ± SD*	*13.0 ± 1.1*
	*Min–Max*	*11.1–14.7*
	PS—2 samples	
	Mean ± SD	11.8 a ± 0.2
	Min–Max	11.6–11.9
	MS—4 samples	
	Mean ± SD	12.0 a ± 0.8
	Min–Max	11.1–13.0
	UHTS—14 samples	
	Mean ± SD	13.5 b ± 0.9
	Min–Max	11.7–14.7

PW = pasteurized whole milk; MW = microfiltered whole milk; UHTW = UHT whole milk; PS= pasteurized semi-skimmed milk; MS = microfiltered semi-skimmed milk; UHTS = UHT semi-skimmed milk; Within the same categories (whole or semi-skimmed milk), values in the same column with different lowercase letters are significantly different (*p* < 0.05).

Although consistent information is not available on the effects of cooking, storage and processing on the choline content of food, these data showed that there were significant differences (*p* < 0.05) in technologically treated milk (UHT *vs.* pasteurized milk and UHT *vs.* microfiltered milk), but not between pasteurized *vs.* microfiltered milk (*p* > 0.05), both in whole and in semi-skimmed milk (Table 3). The milk contains a high concentration of choline, present as free choline, but also in the esterified forms, phosphocholine, phosphatidylcholine, glycerophosphocholine and sphingomyelin [43], classes of phospholipid that are abundant in cell membranes. According to several authors [47,48,49], the phospholipid concentration in milk depends on different stages of lactation or seasonal changes; furthermore, the thermal treatments and other processing techniques could also influence these compounds. To some extent, this variability in phospholipids could justify the results in the choline content of the studied samples.

Moreover, it is worth noting that choline showed species-specific differences: according to the results of other research [50], goat’s milk showed less content of choline than cow’s milk (5.5 mg choline/100 g goat’s milk), regardless of skimming or technological treatments. It would be advisable to extend the investigation on other animal species’ milk (sheep, ewe, donkey, *etc*.).

### 3.4. Unsaponifiable Compounds and Tracing Parameters

In Table 4, the contents of unsaponifiable molecules (cholesterol, β-carotene, α-tocopherol and retinol’s isomers) are shown: as expected, these components showed significant differences (*p* < 0.05) between whole and semi-skimmed milk, because skimmed products contain less fat and cholesterol, but also some other lipid compounds (such as vitamin A and E).

**Table 4:** Cholesterol (mg/100 g), β-carotene, α-tocopherol, 13 *cis* and *trans* retinol (μg/100 g), in whole and semi-skimmed commercial Italian milk. Data are shown as the mean with standard deviation (SD) and minimum and maximum values.

Milk		Cholesterol	β-Carotene	α-Tocopherol	13 *cis* retinol	*trans* retinol
*Whole—18 samples*						
	*Mean ± SD*	*12.8 a ± 0.4*	*7.9 a ± 3.5*	*87.2 a ± 8.8*	*3.3 a ± 2.2*	*46.2 a ± 9.9*
	*Min–Max*	*12.1–13.5*	*4.2–15.3*	*66.2–105.9*	*0.4–7.3*	*31.2–64.4*
	PW—3 samples					
	Mean ± SD	13.1 ± 0.1	5.2 ± 0.9	80.4 ± 3.8	0.7 ^A^ ± 0.3	54.7 ^A^ ± 8.6
	Min–Max	13.0–13.2	4.4–6.2	76.9–84.4	0.4–0.9	44.9–61.0
	MW—3 samples					
	Mean ± SD	12.7 ± 0.2	5.5 ± 1.1	83.4 ± 14.9	1.2 ^A^ ± 0.3	60.0 ^A^ ± 5.8
	Min–Max	12.5–12.9	4.2–6.3	66.2–92.5	0.9–1.5	53.4–64.4
	UHTW—14 samples					
	Mean ± SD	12.8 ± 0.5	9.2 ± 3.6	89.9 ± 7.2	4.4 ^B^ ± 1.7	40.6 ^B^ ± 5.1
	Min–Max	12.1–13.5	5.4–15.3	81.7–105.9	1.9–7.3	31.2–48.0
*Semi-skimmed—20 samples*						
	*Mean ± SD*	*7.0 b ± 0.2*	*4.0 b ± 1.4*	*40.2 b ± 5.9*	*2.1 b ± 1.4*	*21.0 b ± 5.0*
	*Min–Max*	*6.4–7.4*	*2.2–7.1*	*30.4–53.0*	*0.3–4.3*	*14.7–32.5*
	PS—2 samples					
	Mean ± SD	7.1 ± 0.2	2.4 ± 0.0	34.8 ± 3.0	0.5 ^A^ ± 0.0	25.6 ^A^ ± 0.0
	Min–Max	7.0–7.2	2.4–2.5	32.7–36.9	0.4–0.5	25.6–25.7
	MS—4 samples					
	Mean ± SD	7.2 ± 0.2	3.1 ± 0.6	44.2 ± 9.7	0.5 ^A^ ± 0.1	28.0 ^A^ ± 3.5
	Min–Max	7.0–7.4	2.2–3.6	30.5–53.0	0.3–0.6	24.1–32.5
	UHTS—14 samples					
	Mean ± SD	7.0 ± 0.2	4.4 ± 1.4	39.8 ± 4.3	2.8 ^B^ ± 1.0	18.4 ^B^ ± 2.9
	Min–Max	6.4–7.3	3.0–7.2	32.6–49.4	1.3–4.3	14.7–23.8

PW = pasteurized whole milk; MW= microfiltered whole milk; UHTW = UHT whole milk; PS= pasteurized semi-skimmed milk; MS = microfiltered semi-skimmed milk; UHTS = UHT semi-skimmed milk; Values in the same column with different lowercase letters are significantly different (*p* < 0.05); Within the same categories (whole or semi-skimmed milk), values in the same column with different superscript capital letters are significantly different (*p* < 0.05).

Among different technologically treated samples, no significant differences (*p* < 0.05) were detected for cholesterol, β-carotene and α-tocopherol between UHT, pasteurized and microfiltered milk, while 13 *cis* retinol and *trans* retinol showed differences (pasteurized *vs.* UHT milk and microfiltered *vs.* UHT milk; *p* < 0.05). Probably, these differences could be due to the vitamin A content in milk influenced overall by the pasture, seasons and lactation stage and by genetic differences between cows [51,52,53], but in these commercial samples, these features were unknown. 

**Figure 1:**
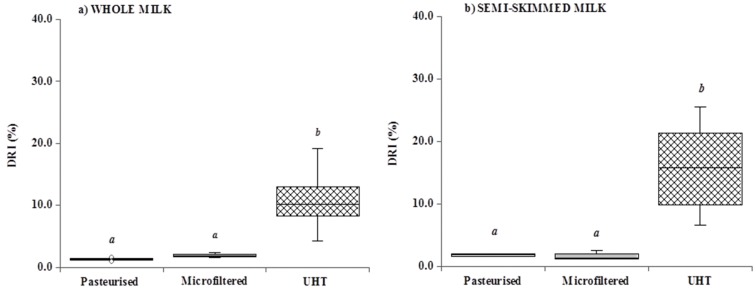
Degree of retinol isomerization (DRI %) in whole (**a**) and semi-skimmed (**b**) commercial milk samples (pasteurized, microfiltered and UHT milk). Within the same categories (whole and semi-skimmed), different superscript letters are significantly different (*p* < 0.05).

It is generally recognized that in milk, vitamin A is heat-resistant; nevertheless, during technological processes (such as heat, light, low pH), some isomerization reactions may occur [54,55]: in particular, *trans* retinol can isomerize to a less potent *cis* form [56]. This is very important overall in nutritional studies, because 13 *cis* retinol has less biopotency (75%) than *trans* retinol (100%) [57]. In Figure 1a,b, the degree of retinol isomerization (DRI), a parameter related to the “severity” of different processing techniques, was shown. In this research, the data confirmed early data [23]: the greater the temperature of milk treatment, the higher the isomerization of retinol.

According to these results, in processed milk, concerning either whole (Figure 1a) or semi-skimmed milk (Figure 1b), there were significant differences (*p* < 0.05) in DRI: in particular, UHT > microfiltration and UHT > pasteurization. 

Moreover, only within UHT milk samples, DRI was different (*p* < 0.05) between UHT whole milk *vs.* UHT semi-skimmed milk (10.9% and 15.4%, respectively). 

**Figure 2:**
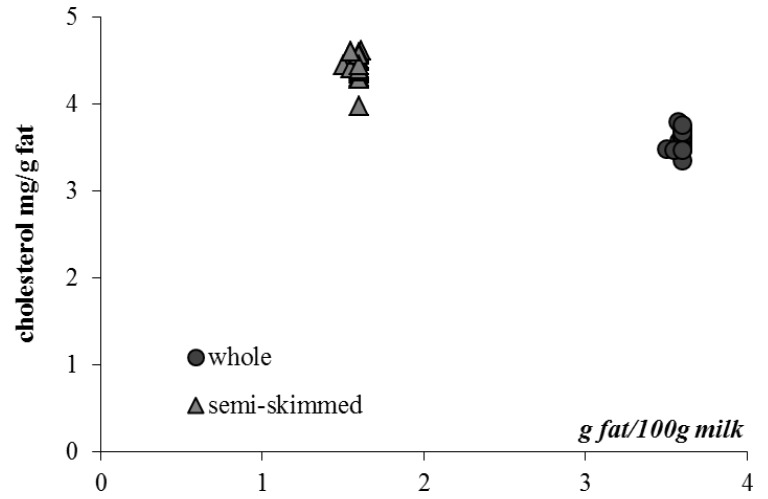
Cholesterol content (mg/g fat) *vs*. fat content (g/100 g) in whole and semi-skimmed commercial milk samples.

All the unsaponifiable data confirmed previous research [58]: as it is well known, during the skimming procedure, the resulting products contain less fat, less cholesterol and less fat-soluble vitamins than whole products. However, this skimming process mainly induces a depletion of the largest cholesterol-poor fat globules [59], and in skimming products, the remaining smallest globules are cholesterol-rich. In Figure 2, the cholesterol, expressed as mg/g of fat, was plotted *vs*. fat content (g/100 g in the studied milk): in semi-skimmed milk the cholesterol level is higher than that in whole milk.

Using the analytical data of this work, the degree of antioxidant protection (DAP) parameter was calculated. The DAP parameter is the molar ratio between antioxidant compounds (β-carotene and α-tocopherol) and a selected oxidation target (cholesterol), and it is a useful parameter to estimate the potential oxidative stability of fat in foods [24,60].

**Figure 3:**
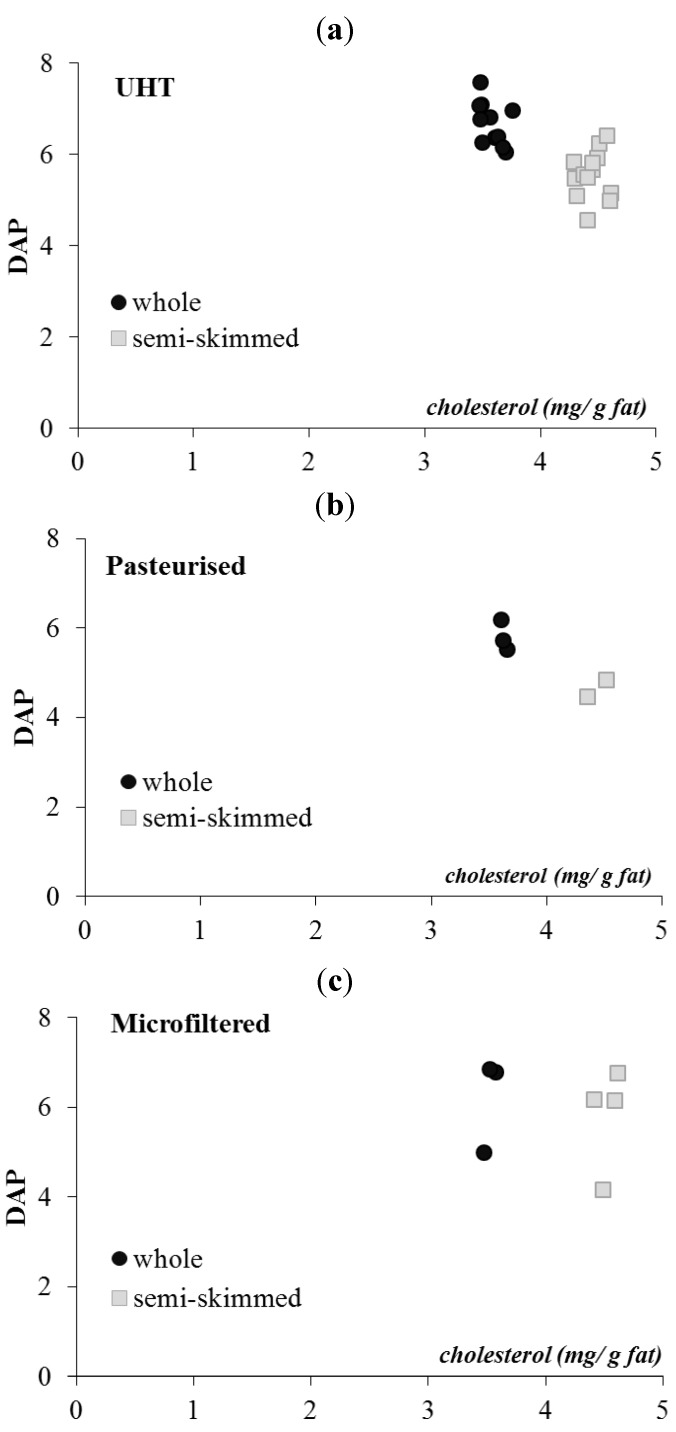
Degree of antioxidant protection (DAP) *vs.* cholesterol content (mg/g fat) in Whole and semi-skimmed commercial UHT (**a**), pasteurized (**b**) and microfiltered (**c**) milk samples.

According to Pizzoferrato and Manzi [58], the cholesterol in skimmed dairy products is more susceptible to oxidation than that of whole fat products, due to the change in the fat antioxidant equilibrium. In Figure 3, cholesterol (mg/g fat) was related to the degree of antioxidant protection (DAP) in studied milk, and the data confirmed that the residual cholesterol in skimmed products is less protected, with a low DAP value, against oxidative agents than the cholesterol in whole dairy products: DAP values were different between whole milk and semi-skimmed milk (*p* < 0.05).

However, within the same technological treatments, these differences (*p* < 0.05) were in UHT milk (Figure 3a) and pasteurized milk (Figure 3b), but not (*p* > 0.05) in microfiltered milk (Figure 3c). This is probably due to the microfiltration treatment: milk fat is removed by centrifugation and totally skimmed milk is forced through the membrane to separate bacteria. Finally, milk was added with a required amount of cream, and it is pasteurized and packaged. Therefore, it is possible that the microfiltration treatment does not determine differences in fat globule sizes between whole and skimmed milk.

### 3.5. Color Measurement

Finally, only in whole milk, the color was determined instrumentally using a colorimeter, where the CIELAB coordinates (L*, a*, b*) were directly read. 

Usually, color measurement of food products is used as an indirect measure of other quality attributes, such as contents of antioxidant compounds, due to its simplicity and quickness, and this measure correlates with other physicochemical properties. The parameter, a*, takes positive values for red colors and negative values for the green ones, whereas b* takes positive values for yellow colors and negative values for the blue ones [61,62], and L* is an index of lightness.

**Figure 4:**
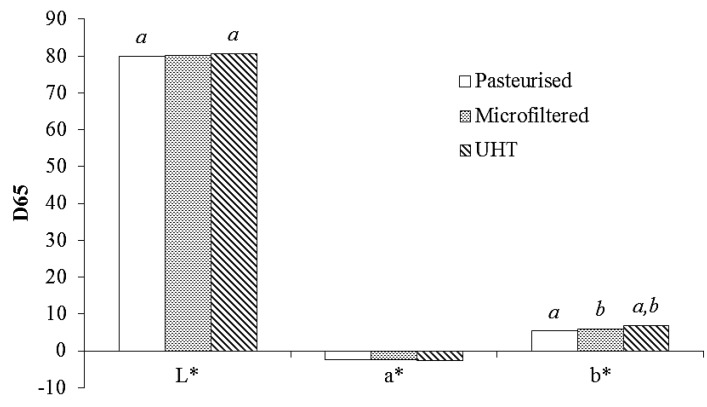
Instrumental determination of color (L*, a*, b*) in whole commercial milk samples (pasteurized, microfiltered and UHT milk). Different superscript letters are significantly different (*p* < 0.05).

According to Popov-Raljić *et al*. [63], storage, the temperature of processing and chemical reactions, such as Maillard’s reaction, represented physico-chemical changes able to influence milk color. The measurements of color parameters (Figure 4) in the studied whole milk samples confirmed their results: L*, index of lightness, was different (*p* < 0.05) between UHT *vs.* pasteurized milk, and the b* value was different between UHT *vs.* pasteurized milk and between UHT *vs.* microfiltered milk. Moreover, the obtained data of whole milk indicated that, among the unsaponifiable molecules, β-carotene content was well correlated with b* (*r* = 0.854), while among the other molecules, lactulose was well correlated with a* (*r* = 0.862). 

## 4. Conclusions

In this study, the nutritional characteristics of UHT, pasteurized and microfiltered milk, whole and semi-skimmed milk, commercialized in Italy from 2009 to 2012, were evaluated.

Calcium and sodium contents did not show differences among milk samples, while only in phosphorus content, significant differences were observed between whole and semi-skimmed milk. Significant differences were observed for lactose, (pasteurized *vs.* UHT milk; *p* < 0.05), and particularly, in severe heat treated milk (UHT), lactose isomerization occurred and lactulose (ranged from 8.6 to 104.0 mg/100 g) was detected. Moreover, the color measurement of whole milk showed a good correlation between lactulose and a* (*r* = 0.862).

There were significant differences (*p* < 0.05) in technologically treated milk (UHT *vs.* pasteurized milk and UHT *vs.* microfiltered milk) in choline content.

This research confirmed that the greater the “severity” of milk treatment, the higher the degree of retinol isomerization. The degree of antioxidant protection (DAP), useful to estimate the potential oxidative stability of fat in foods, was significantly different between whole and semi-skimmed milk, and mainly, these differences were in UHT milk (*p* < 0.05) and pasteurized milk (*p* < 0.05), but not in microfiltered milk (*p* > 0.05). The data showed that the residual cholesterol in skimmed products was less protected against oxidative agents than the cholesterol in whole dairy products, but within the same technological treatments, these differences were only in UHT and pasteurized milk.

## References

[1] International Dairy Federation (2010). The World Dairy Situation 2010. Bulletin of the International Dairy Federation 446/2010.

[2] Gerosa S., Skoet J. (2012). Milk Availability: Trends in Production and Demand and Medium-Term Outlook. http://www.fao.org/docrep/015/an450e/an450e00.pdf.

[3] OECD-FAO Agricultural Outlook 2012–2021. http://dx.doi.org/10.1787/agr_outlook-2012-en.

[4] Italian Law Ministero della Salute, Decreto 12/12/2012. http://www.trovanorme.salute.gov.it/dettaglioAtto?id=45076&completo=true.

[5] Konosonoka I.H., Jemeljanovs A., Osmane B., Ikauniece D., Gulbe G. (2012). Incidence of *Listeria* spp. in dairy cows feed and raw milk in Latvia. ISRN Vet. Sci..

[6] Hunt K., Drummond N., Murphy  M., Butler F., Buckley J., Jordan K. (2012). A case of bovine raw milk contamination with *Listeria* monocytogenes. Ir. Vet. J..

[7] Claeys W.L., Cardoen S., Daube G., de Block J., Dewettinck K., Dierick K., de Zutter L., Huyghebaert A., Imberechts H., Thiange P. (2013). Raw or heated cow milk consumption: Review of risks and benefits. Food Control.

[8] Oliver S.P., Boor K.J., Murphy S.C., Murinda S.E. (2009). Food safety hazards associated with consumption of raw milk. Foodborne Pathog. Dis..

[9] Council Directive 92/46/EEC of 16 June 1992 Laying down the Health Rules for the Production and Placing on the Market of Raw Milk, Heat-Treated Milk and Milk-Based Product. http://ec.europa.eu/food/fs/sfp/mr/mr03_en.pdf.

[10] Italian Law Ministero della salute, Decreto 17/6/2002. http://gazzette.comune.jesi.an.it/2002/178/6.htm.

[11] Garcia Fernandez L., Alvarez Blanco S., Riera Rodrıguez F.A. (2013). Microfiltration applied to dairy streams: Removal of bacteria. J. Sci. Food Agric..

[12] Saboya L.V., Maubois J.L. (2000). Current developments of microfiltration technology in the dairy industry. Lait.

[13] Elwell M.W., Barbano D.M. (2006). Use of microfiltration to improve fluid milk quality. J. Dairy Sci..

[14] Haug A., Høstmark A.T., Harstad O.M. (2007). Bovine milk in human nutrition—A review. Lipids Health Dis..

[15] Van Boekel M.A.J.S. (1998). Effect of heating on Maillard reactions in milk. Food Chem..

[16] O’Connell J.E., Fox P.F., Roginski H., Fuquay J.W., Fox P.F. (2002). O’Connell, J.E.; Fox, P.F. Heat Stability of Milk. Encyclopedia of Dairy Sciences.

[17] Seiquer I., Delgado-Andrade C., Haro A., Navarro M.P. (2010). Assessing the effects of severe heat treatment of milk on calcium bioavailability: *In vitro* and *in vivo* studies. J. Dairy Sci..

[18] (2002). AOAC Official Methods of Analysis of the Association of Official Analytical Chemists. Method 991.25. Calcium, Magnesium and Phosphorus in Cheese.

[19] Indyk H.E., Edwards M.J., Woollard D.C. (1996). High performance liquid chromatographic analysis of lactose-hydrolysed milk. Food Chem..

[20] Manzi P., Pizzoferrato L. (2013). HPLC determination of lactulose in heat treated milk. Food Bioprocess Technol..

[21] (2000). AOAC Official Methods of Analysis of the Association of Official Analytical Chemists. Method 999.14. Choline in Infant Formula and Milk.

[22] Panfili G., Manzi P., Pizzoferrato L. (1994). High performance liquid chromatographic method for the simultaneous determination of tocopherols, carotenes, and retinol and its geometric isomers in Italian cheeses. Analyst.

[23] Panfili G., Manzi P., Pizzoferrato L. (1998). Influence of thermal and other manufacturing stresses on retinol isomerization in milk and dairy products. J. Dairy Res..

[24] Pizzoferrato L., Manzi P., Marconi S., Fedele V., Claps S., Rubino R. (2007). Degree of antioxidant protection: A parameter to trace the origin and quality of goat’s milk and cheese. J. Dairy Sci..

[25] Gaucheron F. (2005). The minerals of milk. Reprod. Nutr. Dev..

[26] Cashman K.D. (2006). Milk minerals including trace elements and bone health. Int. Dairy J..

[27] Hidiroglou M., Proulx J.G. (1982). Factors affecting the calcium, magnesium and phosphorus content of beef cow milk. Can. J. Comp. Med..

[28] Miller G.D., di Rienzo D.D., Reusser M.E., McCarron D.A. (2000). Benefits of dairy product consumption on blood pressure in humans: A summary of the biomedical literature. J. Am. Coll. Nutr..

[29] Zemel M.B. (2003). Role of dietary calcium and dairy products in modulating adiposity. Lipids..

[30] Fulgoni V.L., Huth P.J., di Rienzo D.B., Miller G.D. (2004). Determination of the optimal number of dairy servings to ensure a low prevalence of inadequate calcium intake in Americans. J. Am. Coll. Nutr..

[31] LARN (1996). Livelli di Assunzione Raccomandati di Energia e Nutrienti per la Popolazione Italiana.

[32] Stephens A.M., Haddad A.C., Phillips S.J. (1983). Passage of carbohydrates into the colon. Gastroenterology.

[33] Adam A.C., Rubio-Texeira M., Polaina J. (2004). Lactose: The milk sugar from a biotechnological perspective. Crit. Rev. Food Sci. Nutr..

[34] Siddique F., Anjum F.M., Huma N., Jamil A. (2010). Effect of different UHT processing temperatures on ash and lactose content of milk during storage at different temperatures. Int. J. Agric. Biol..

[35] Berning L.M., Shook G.E. (1992). Prediction of mastitis using milk somatic cell count, N-acetyl-beta-d-glucosaminidase and lactose. J. Dairy Sci..

[36] Morales F.J., Romero C., Jimenez-Perez S. (2000). Characterization of industrial processed milk by analysis of heat-induced changes. Int. J. Food Sci. Technol..

[37] López-Fandiño R., Olano A. (1999). Review: Selected indicators of the quality of thermal processed milk. Food Sci. Technol. Int..

[38] Mortier L., Braekman A., Cartuyvels D., van Renterghem R., de Block J. (2000). Intrinsic indicators for monitoring heat damage of consumption milk. Biotechnol. Agron. Soc. Environ..

[39] Schuck P. (2002). Spray drying of dairy products, state of the art. Lait.

[40] Roberfroid M. (1999). Concepts in functional foods: The case of inulin and oligofructose. J. Nutr..

[41] Milner J.A. (1999). Functional foods and health promotion. J. Nutr..

[42] Zeisel S.H., Blusztajn J.K. (1994). Choline and human nutrition. Annu. Rev. Nutr..

[43] Holmes-McNary M.Q., Cheng W.L., Mar M.H., Fussel S., Zeisel S.H. (1996). Choline and choline esters in human and rat milk and infant formulas. Am. J. Clin. Nutr..

[44] Zeisel S.H., Mar M.H., Howe J.C., Holden J.M. (2003). Concentrations of choline-containing compounds and betaine in common foods. J. Nutr..

[45] Li Z., Vance D.E. (2008). Phosphatidylcholine and choline homeostasis. J. Lipid Res..

[46] Rajaie S., Esmaillzadeh A. (2011). Dietary choline and betaine intakes and risk of cardiovascular diseases: Review of epidemiological evidence. ARYA Atheroscler..

[47] Christie W.W., Noble R.C., Davies G. (1987). Phospholipids in milk and dairy-products. J. Soc. Dairy Technol..

[48] Bitman J., Wood D.L. (1990). Changes in milk-fat phospholipids during lactation. J. Dairy Sci..

[49] Rombaut R., Dewettinck K., van Camp J. (2007). Phospho- and sphingolipid content of selected dairy products as determined by HPLC coupled to an evaporative light scattering detector (HPLC-ELSD). J. Food Comp. Anal..

[50] Mattera M., Manzi P., Pizzoferrato L. (2011). Colina e Lattulosio nel Latte di Origine Vaccina e Caprina. Proceedings of VIII Congresso Nazionale di Chimica degli Alimenti—Qualità e Tipicità Degli Alimenti Mediterranei: Alimentazione e Salute, Messina 20–24/9/2010.

[51] Noziere P., Graulet B., Lucas A., Martin B., Grolier P., Doreau M. (2006). Carotenoids for ruminants: From forages to dairy products. Anim. Feed Sci. Technol..

[52] Noziere P., Grolier P., Durand D., Ferlay A., Pradel P., Martin B. (2006). Variations in carotenoids, fat-soluble micronutrients, and colour in cows’ plasma and milk following changes in forage and feeding level. J. Dairy Sci..

[53] Jensen S.K., Johannsen A.K.B., Hermansen J.E. (1999). Quantitative secretion and maximal secretion capacity of retinol, β-carotene and α-tocopherol into cows’ milk. J. Dairy Res..

[54] Panfili G., Fratianni A., di Criscio T., Gammariello D., Sorrentino E. (2008). Influence of microorganisms on retinol isomerization in milk. J. Dairy Res..

[55] Marconi E., Panfili G. (1998). Chemical composition and nutritional properties of commercial products of mare milk powder. J. Food Comp. Anal..

[56] Erdman J.W., Poor C.L., Dietz J.M. (1988). Factors affecting the bioavailability of vitamin A, carotenoids, and vitamin E. Food Technol..

[57] Weiser H., Somorjai G. (1992). Bioactivity of *cis* and *dicis* isomers of vitamin A esters. Int. J. Vitam. Nutr. Res..

[58] Manzi P., Pizzoferrato L. (2010). Cholesterol and antioxidant vitamins in fat fraction of whole and skimmed dairy products. Food Bioprocess Technol..

[59] Cerutti G., Machado M.A., Ribalzi L. (1993). Sulla distribuzione del colesterolo in latte e derivati. Latte.

[60] Esti M., Cinquanta L., Panfili G., Manzi P., Marconi S., Pizzoferrato L. (2004). Effects of variety and olive ripeness on nutritional quality and oxidative stability of extra virgin olive oils. Food Agric. Environ..

[61] Pathare P.B., Opara U.L., Al-Said F.A. (2013). Colour measurement and analysis in fresh and processed foods: A review. Food Bioprocess Technol..

[62] Barrett D.M., Beaulieu J.C., Shewfelt R. (2010). Colour, flavor, texture, and nutritional quality of fresh-cut fruits and vegetables: Desirable levels, instrumental and sensory measurement, and the effects of processing. Crit. Rev. Food Sci. Nutr..

[63] Popov-Raljić J.V., Lakić N.S., Laličić-Petronijević J.G., Barać M.B., Sikimić V.M. (2008). Colour changes of UHT milk during storage. Sensors.

